# Evaluation of 2,3-butanediol derived from whey fermentation as an effective bio-based monomer for waterborne polyurethane dispersions

**DOI:** 10.3389/fchem.2024.1516427

**Published:** 2025-01-06

**Authors:** Lorena Germán-Ayuso, Rubén Cobos, Leire Lorenzo, Francisca Río, Soraya Prieto-Fernández, Tomás Roncal, José M. Cuevas

**Affiliations:** ^1^ GAIKER Technology Centre, Basque Research and Technology Alliance (BRTA), Parque Tecnológico de Bizkaia, Zamudio, Spain; ^2^ TECNALIA, Basque Research and Technology Alliance (BRTA), Parque Tecnológico de Álava, Miñano, Spain; ^3^ TECNALIA, Basque Research and Technology Alliance (BRTA), Parque Tecnológico de San Sebastián, Donostia-San Sebastián, Spain

**Keywords:** whey, fermenation, 2,3-butanediol, polyurethane dispersions, water based coatings, biobased polyurethanes

## Abstract

Within the context of the circular economy, the transformation of agri-food waste or by-products into valuable products is essential to promoting a transition towards more sustainable and efficient utilisation of resources. Whey is a very abundant by-product of dairy manufacturing. Apart from partial reutilisation in animal feed or some food supplements, the sustainable management and disposal of whey still represent significant environmental challenges. In this work, whey is considered a valuable resource for producing high-value products, specifically 2,3-butanediol (2,3-BDO), which was produced through fermentation using the bacterial strain *Lactococcus lactis* 43103. The described process yielded a >90% purity of 2,3-BDO, which was evaluated as a potential chain extender in the synthesis of bio-based waterborne polyurethane dispersions (PUDs). The incorporation of whey-derived 2,3-BDO led to the development of PUDs with up to 90% bio-based content without detrimental effects on the process or liquid-phase properties. The combination of 100% bio-based polyether polyols with partially renewable L-lysine ethyl ester diisocyanate and whey-derived 2,3-BDO as a chain extender generated totally stable, low-particle-size water dispersions of amorphous polymers characterised by similar structure and molecular weight compared to those of alternative petroleum-based PUDs. These results open up the possibility of incorporating fermentation-derived 2,3-BDO as a totally renewable component in bio-based PUDs as potential sustainable resinous systems for further formulation of water-based coatings or adhesives.

## 1 Introduction

The increasing demand for chemicals in personal and industrial applications has a significant impact on the environment, largely due to fossil-dependent large-scale production (Ramchuran et al., 2023). Thus, the chemical industry requires alternative pathways to produce functional chemicals by shifting from fossil-based feedstock to materials of biological origin. Those biochemicals can play a key role within a global bioeconomy strategy, where their future expansion as viable alternatives to conventional chemical sources depends on many factors. In addition to biomass availability, efficient production, cost, safety, and sustainability, a key requirement is the ability to perform effectively in multi-step processing and product chains.

2,3-Butanediol (2,3-BDO) is a platform chemical with many industrial applications (Xie et al., 2022), including its use as a building block in the manufacture of a wide range of chemicals and polymers. It can be used as a solvent, antifreeze agent, liquid fuel, and monomer for the manufacture of many synthetic polymers and resins. 2,3-BDO also finds additional potential applications in the production of printing inks, perfumes, fumigants, spandex, moistening and softening agents, plasticisers and as a carrier for pharmaceuticals. Of special relevance are the uses of 2,3-BDO as a building block, that is, as a precursor in the synthesis of valuable industrial chemicals, including acetoin and diacetyl, methyl ethyl ketone, 2-butanol, butene, and 1,3-butadiene, and as a monomer in the manufacture of some types of polymers, such as polyesters, polycarbonates, and polyurethanes.

Currently, the industrial production of 2,3-BDO mainly relies on petrochemical feedstocks through a complex and costly process (Gräfje et al., 2019). Owing to these concerns, trends are shifting towards the search for more environmentally friendly and cost-competitive alternatives, and among them, the biological production of 2,3-BDO stands out as an outstanding option. Many microorganisms are known to naturally produce 2,3-BDO (Xie et al., 2022), including *Lactococcus lactis* (Roncal et al., 2017a; Roncal et al., 2023), a non-pathogenic and GRAS (generally recognised as safe) microorganism. In addition to the *per se* more sustainable biological production of 2,3-BDO, the use of bio-based residual streams or industrial by-products as feedstock would add more value to and support the circularity of the process.

In recent years, waterborne polyurethane dispersions (PUDs) have been widely used as very versatile, safe, and non-toxic polymeric materials for coatings, adhesives, and inks. The numerous tailor-made structural features in these VOC-free (VOC, volatile organic compound) resinous systems are achievable from selected components and designed interactions between the hard and soft segments in polyurethane. Nevertheless, PUD technology still relies on petrol-based raw materials, which prevent enhanced sustainability. Therefore, in a quest to replace petrochemical components, increasing interest in validating bio-based alternative components like polyols, isocyanates, or chain extenders in PUD technology is continuously growing (Xue et al., 2024; De Smet et al., 2021; Liang et al., 2018; Zhang et al., 2019).

PUDs are colloidal systems with polyurethane particles dispersed in aqueous media due to internal emulsifiers within a linear polyurethane prepolymer. The target high-performance polyurethane is subsequently obtained by extending this isocyanate-terminated prepolymer with low molecular weight alcohols or amines (Germán-Ayuso et al., 2022). In this scenario, it is extremely necessary to thoroughly evaluate the suitability of any bio-based alternative component before formulating a water-based coating. This assessment considers not only the synthesis of the polyurethane but also the dispersion of the polymer in water and the associated properties of the liquid resinous PUDs, like particle size and stability of the emulsion (Qi et al., 2017).

Following this major trend and necessity of looking for alternative renewable chemicals to develop new bio-based PUDs—preferably derived from renewable residual streams or by-products within promoted circularity (De Smet et al., 2021; Yin et al., 2024)—this study explores the preparation of 2,3-BDO from whey fermentation as a promising diol in waterborne PU chemistry. Thus, this renewable building block was assessed as a potential chain extender in enhancing bio-based content PUDs, considering the key properties of the liquid polyurethane dispersions. The work promoted the bio-based content through the innovative integration of bio-based polyol with a renewable co-solvent and isocyanate during prepolymer synthesis, before further extension with fermentation-derived 2,3-BDO. Then, the effective substitution of petrol-based solvents with dihydrolevoglucosenone as a reaction co-solvent, demonstrated by Germán et al. (2021), was combined, for the first time, with L-Lysine ethyl ester diisocyanate to design a PUD with over 80% bio-based content when bio-based 2,3-BDO was used as suitable chain extender. Therefore, the obtained results demonstrated the potential substitution of petrol-based counterparts with this renewable diol, increasing the bio-based content in equivalent and stable colloidal systems without compromising the reaction process, polymer structure, or key liquid-phase properties.

## 2 Materials and procedures

### 2.1 Production of bio-based 2,3-BDO by fermentation

Bio-based 2,3-BDO was produced by fermentation using the 2,3-BDO-overproducing mutant strain *L. lactis* 43103 (Roncal et al., 2017a) in a whey-based medium as previously described by Roncal et al. (2023). The whey-based culture medium (WCSLCP) contained 73 g L^−1^ spray-dried whey from bovine milk (Sigma), 20 g L^−1^ corn steep liquor, 5 g L^−1^ casein peptone, and 0.5 mL L^−1^ antifoam 204 (Sigma). This concentration of whey resulted in a lactose concentration close to 50 g L^−1^, a value similar to that found in fresh liquid whey. For WCSLCP media preparation, following autoclave sterilisation, the proteins found naturally in whey were hydrolysed through the sequential treatment with two commercial protease preparations, Alcalase 2.4 L and Flavourzyme (both from Novozymes), at a concentration rate of enzyme: medium of 1:1000 (v/v). First, the medium was treated with Alcalase 2.4 L for 2 h at pH 8.2, 50°C, and 500 rpm, followed by treatment for another 2 h with Flavourzyme at pH 7.0 and the same temperature and stir rate. The previous step of hydrolysing the whey proteins was included to generate the free amino acids and oligopeptides that are assimilable by the strain.

Fermentations, aerobic and in batch mode, were carried out in a Biostat B-plus fermenter (Sartorius) equipped with a 2-L reaction vessel containing 1 L of the culture medium. Fermentations were carried out at 30°C, pH 6.0, and 10% dissolved oxygen concentration (DOC), expressed as % with respect to the oxygen saturation concentration in the medium. Medium pH was kept constant by the automatic addition of sodium hydroxide (5 M NaOH). DOC was controlled through a cascade control strategy including sequential steps of stir rate increase, oxygen enrichment, and gas flow increase. The basal values for these parameters were 150 rpm, 21% (the oxygen content in air), and 0.25 L L^−1^ min^−1^ and could be increased sequentially to 500 rpm, 50%, and 1 L L^−1^ min^−1^, respectively, as needed to maintain the DOC at the set point value.

Fermentations were started with an inoculum of 2% (v/v) from a culture of the strain grown in the YEC medium (1.5% glucose, 10 g L^−1^ yeast extract, and 20 mM citrate buffer, pH 7.0) for 24 h at 30°C and 250 rpm orbital shaking.

For a detailed description of the 2,3-BDO fermentation process, please refer to Roncal et al. (2023).

### 2.2 Purification of 2,3-BDO

**FIGURE 1:**
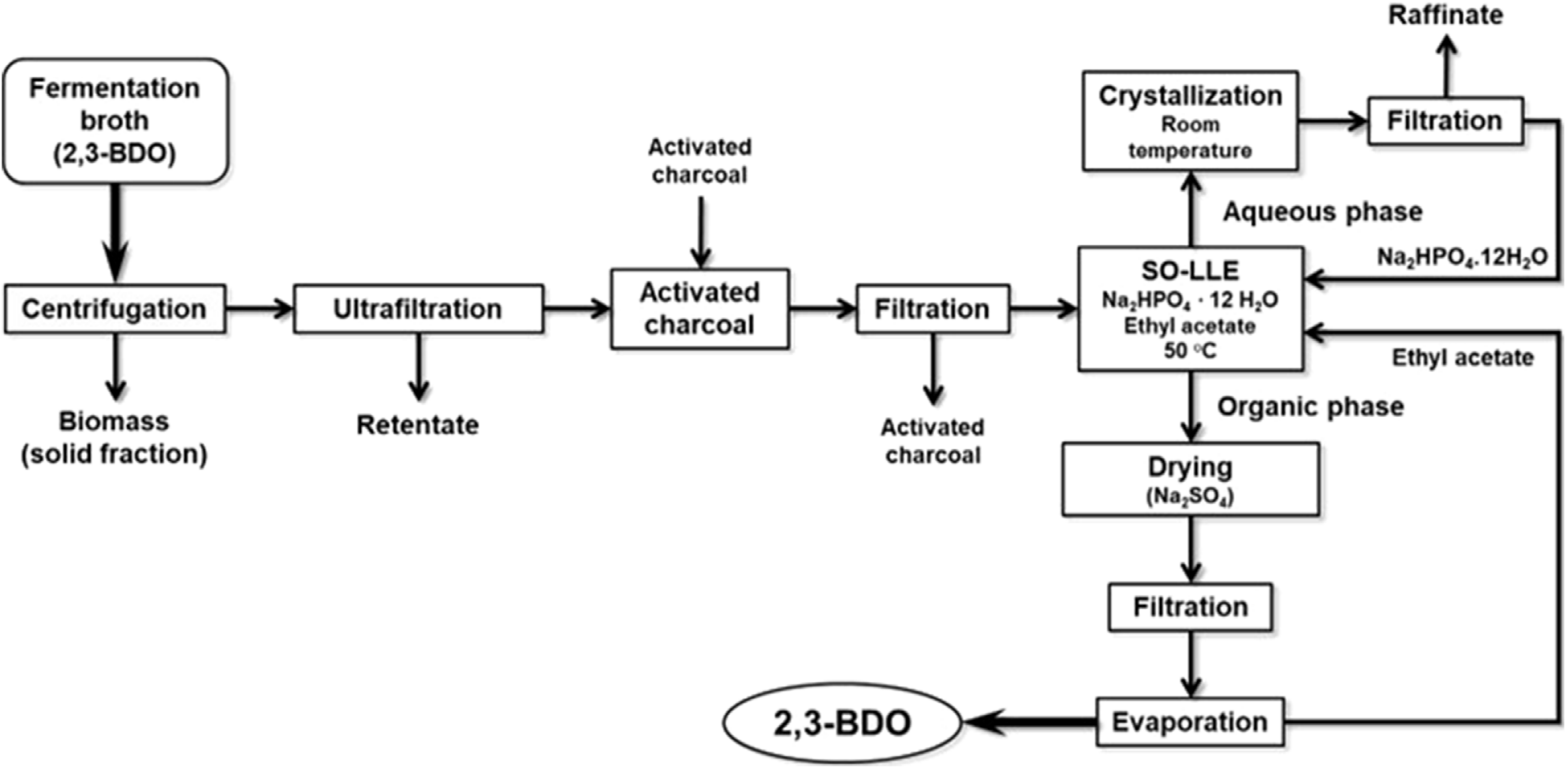
Scheme of the purification process of 2,3-BDO from the fermentation broth.

The purification of 2,3-BDO from fermentation broth was addressed by salting-out liquid–liquid extraction (SO-LLE) in a process involving several sequential steps, as shown in Figure 1. First, the fermentation broth was centrifuged at 4,000 rpm for 30 min to remove the bacterial biomass and other insoluble matter from the 2,3-BDO-containing liquid fraction. The clarified broth was then ultrafiltered to remove high-molecular weight compounds (proteins, biopolymers, etc.) using a Koch UF HFK-131 membrane (MWCO, 10 kDa) at 7 bar pressure and 45°C in a KMS Laboratory Cell CF-1 operating under tangential flow mode. Ultrafiltration was stopped when the volumetric ratio of permeate to retentate was 10. Next, the ultrafiltration permeate was treated with 2.5% (w/w) Clarimex MMF-activated charcoal for 10 min with stirring to remove colouring substances. The activated charcoal was then separated by filtration through a GF/A 1820–150 filter (pore size, 1.6 mm), and the clean liquid passed to the SO-LLE stage. The 2,3-BDO-containing liquid was put into a jacketed extraction vessel equipped with mechanical stirring and a reflux condenser. It was heated to 50°C, and 31.8% (w/w) disodium hydrogen phosphate dodecahydrate (Na_2_HPO_4_ · 12 H_2_O) was dissolved in it. Later, a volume of ethyl acetate enough to reach an organic phase/aqueous phase volumetric ratio of 0.47 was added. The mixture was stirred for 2 min, and then the two phases were allowed to separate for 3 min. The upper 2,3-BDO-enriched organic phase was recovered, and the lower aqueous phase depleted in 2,3-BDO was subjected to a second extraction with the same amount of ethyl acetate and conditions. Both organic phases were pooled and treated for 10 min with 1.5% (w/w) anhydrous sodium sulphate to remove any trace of water. Finally, ethyl acetate was evaporated in a rotary evaporator at 40°C and 200 mbar, leaving purified 2,3-BDO on one side, while recovering the solvent on the other side for reuse in further extractions. The 2,3-BDO-depleted aqueous phase resulting from extraction, containing a high concentration of Na_2_HPO_4_ · 12 H_2_O, was let to cool to room temperature to favour crystallisation of the salt, which could be recovered to be reused.

### 2.3 Determination of metabolite concentrations

The concentrations of 2,3-BDO, acetoin, lactic acid, acetic acid, ethanol, glycerol, and sugars (lactose, glucose, and galactose) were measured by high-performance liquid chromatography (HPLC) using an Agilent Technologies 1260 Infinity Series System, equipped with an Aminex HPX-87H Column (300 × 7.8 mm; Bio Rad) and a Microguard Cation H Refill Cartridge precolumn. The following conditions were used: mobile phase, 0.01 N H_2_SO_4_; flow rate, 0.7 mL min^−1^; and column temperature, 65°C. Peak quantification was performed using a refractive index detector (Agilent G1362A). A calibration curve for 2,3-BDO was prepared in the range of 0.2–30 g L^−1^.

### 2.4 Synthesis of waterborne polyurethane dispersions

**FIGURE 2:**
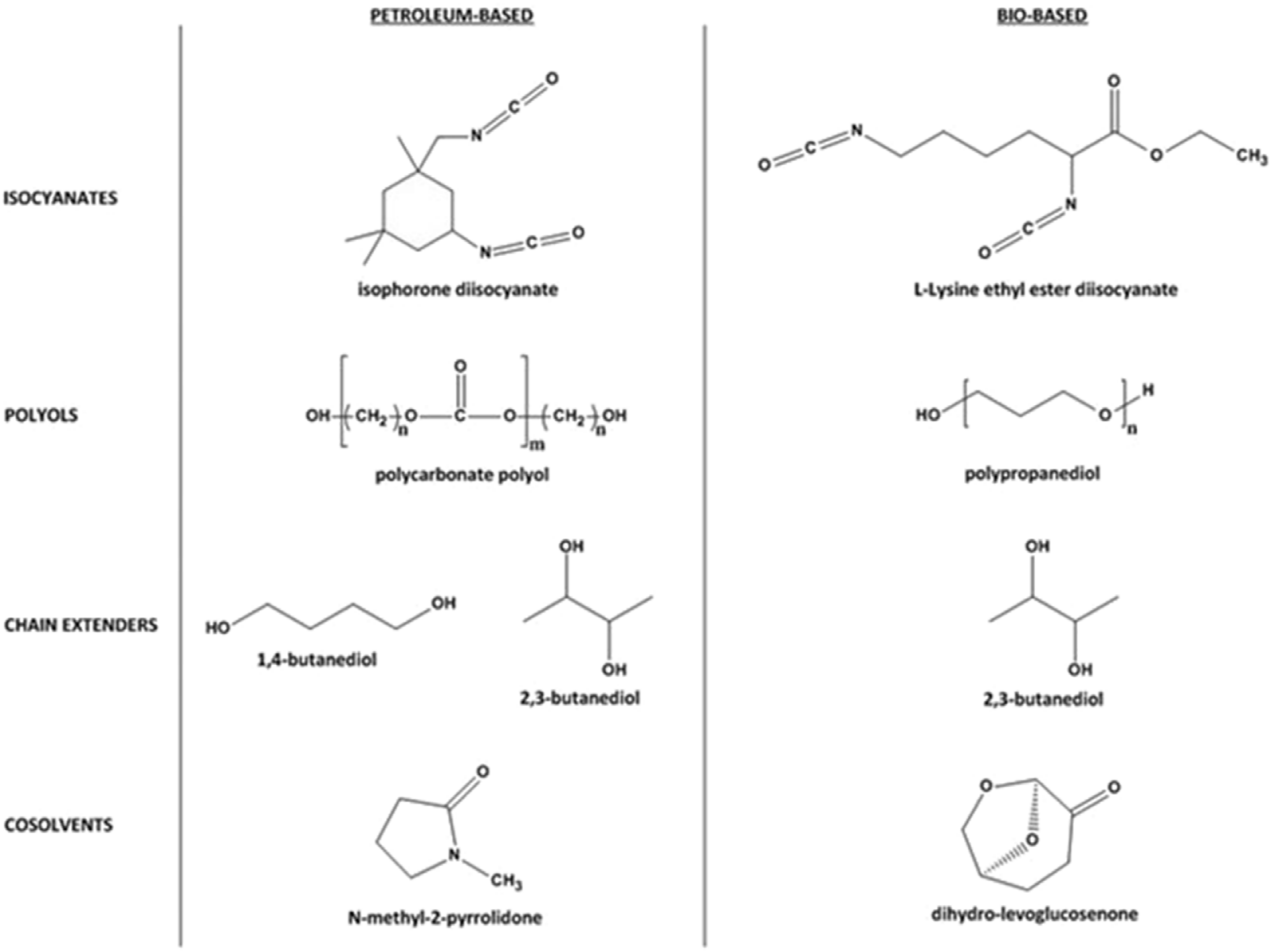
Main components in synthesised polyurethane dispersions.

Three different polyols were used as soft segment components in aqueous polyurethane dispersions. Duranol^TM^ T5651 (1000 g mol^−1^) from Asahi Kasei was selected as a reference petroleum-based polycarbonate polyol, and two different 1,3-propanediol-derived polyether polyols were selected as 100% bio-based alternative soft segment elements. These polypropanediols, based on renewable feedstock, were Velvetol H1000 (1000 g mol^−1^) and Velvetol H500 (500 g mol^−1^) from WeylChem. Dimethylolpropionic acid (DMPA) and dibutyltin dilaurate (DBTDL) (Sigma Aldrich) were used as the internal emulsifier and catalyst, respectively. Triethylamine (TEA) (Sigma Aldrich), 1,4-butanediol (1,4-BDO) (Sigma Aldrich), petroleum-derived 2,3-BDO (Sigma Aldrich), and the whey-derived 2,3-BDO (w2,3-BDO) were the neutraliser and the chain extenders in the polyurethane structures. Isocyanates selected for this study included isophorone diisocyanate (IPDI) from Merck and bio-based L-Lysine ethyl ester diisocyanate (LY) from Thermo Scientific Chemicals. N-methyl-2-pyrrolidone (NMP) (Sigma Aldrich) and bio-based dihydrolevoglucosenone (CY) (Circa) were used as co-solvents (Figure 2).

Polyols, isocyanate, internal emulsifier, catalyst, and co-solvent reacted in the reactor under mechanical stirring at 80°C to obtain an NCO-terminated polyurethane prepolymer, maintaining a 1.5 NCO/OH ratio. After full neutralisation of acid moieties of the prepolymer with TEA at lower temperatures, the chain extender was added to synthesise the polyurethane. Afterwards, water dispersion was achieved by gradual incorporation of distilled water under vigorous stirring.

Two series of PUDs were synthesised to analyse 2,3-BDO as a chain extender. First, the suitability of 2,3-BDO as a chain extender was evaluated by synthesising two petrol-based standard polyurethane formulations with 44 wt% hard segment content: one with the most widely used chain extender 1,4-BDO and the other one with synthetic 2,3-BDO. In addition, the second series was synthesised with the same [NCO]/[BDO] molar ratio but enhanced bio-based content by using polyol, isocyanate, co-solvent, and chain extender from renewable resources. The synthesised PUDs are described in Table 1.

**TABLE 1:** Synthesised polyurethane dispersions.

Polyurethane	Polyol	Isocyanate	Chain extender	Hard segment content (%wt)	Co-solvent (%wt)	Bio-based content (%wt)
PUD-1	T5651	IPDI	1,4-BDO	44	NMP (12)	0
PUD-2	T5651	IPDI	2,3-BDO	44	NMP (12)	0
PUD-3	H1000	LY	w2,3-BDO	44	CY (12)	89.0^b^
PUD-4	H500	LY	w2,3-BDO	55^a^	CY (12)	88.8^b^

^a^ Hard segment content varied from equal [NCO]/[BDO] ratio with a lower molecular weight polyol.

^b^ The % of bio-based material in the polyurethanes was calculated without taking into account the co-solvent.

The conversion of NCO groups during the polyurethane synthesis was monitored by attenuated total reflection equipped with Fourier-transform infrared (ATR-FTIR). Infrared spectra were obtained using a Shimadzu MIRacle 10 Spectrometer and recorded in a range of 4,000–400 cm^−1^, and 20 scans were averaged with a nominal resolution of 4 cm^−1^.

The quantification of the conversion of NCO groups is based on the Beer–Lambert law and calculated from decay in the corresponding absorbance. The area below the isocyanate stretch peak located at ∼2,260 cm^−1^, thus, is measured, and the result is normalised with respect to the area below the C–H stretch peaks between ∼2,840 and ∼3,050 cm^−1^ (Cateto et al., 2008; Tilly et al., 2019). The conversion of the isocyanate functional group as a function of time was calculated from

$$Conversion=1-\frac{A_{t}}{A_{0}},$$

where A_t_ is the normalised area under the isocyanate peak at different reaction times t and A_0_ is the initial normalised area under the isocyanate peak (t = 0).

### 2.5 Characterisation of dispersions

The particle size distribution of the different PUDs was evaluated by measuring variations in the light scattered using the dynamic light scattering (DLS) technique, whereas zeta potential was established by phase analysis light scattering from the electrophoretic mobility of the dispersions. The Z potential provides information about the effectivity of electrostatic stabilisation provided by the internal emulsifier and the concentration of ions in the system. These parameters were measured using the Brookhaven ZetaPALS instrument at 25°C with samples diluted with deionised water.

The analysis of the medium-term stability of PUDs was performed using the Turbiscan™ LAB Stability Analyser (Formulaction SA). The technology was based on the multiple light scattering (MLS) technique, which identifies destabilisation mechanisms in dispersions such as creaming, sedimentation, flocculation, and coalescence. The equipment calculated the Turbiscan^®^ Stability Index (TSI) (Qi et al., 2017), a specific parameter that quantifies and compares the physical stability of samples. This index, thus, can be associated with the destabilisation kinetics by analysing its evolution with time (Qi et al., 2017). In this work, the stability of PUDs was evaluated, without dilution, at different times.

Dry transparent samples were prepared by casting 4 g of polyurethane dispersion in a Teflon mould and allowing evaporation of the water at room temperature for 7 days. Afterwards, polyurethane was dried at 50°C for 24 h. The dried polymers were analysed by attenuated total reflectance - Fourier transform infrared spectroscopy (ATR-FTIR) to evaluate the chemical structure. Furthermore, the average molecular masses were determined by gel permeation chromatography equipped with a refractive index detector 2414 and two columns, TOSOH HR1 and HR3 (GPC WATERS). The equipment was calibrated with polystyrene standards, and 70 mL of the sample dissolved in tetrahydrofuran (THF) was injected with a volume rate of the carrier solvent of 1 mL min^−1^ at 40°C.

The thermal stability and properties of PUDs were evaluated by thermogravimetric (TGA) and calorimetric analysis (DSC) using the Mettler Toledo TGA/DSC 851e Thermobalance. The TGA experiments were performed at a 20°C min^−1^ heating rate from 25°C to 600°C under a nitrogen atmosphere. The DSC tests were conducted under nitrogen flow, first heating the samples from −25°C to 180°C at a rate of 10°C min^−1^. Subsequently, a cooling scan from 180 to −80°C at a rate of −10°C min^−1^ was performed, followed by a second heating scan to 180°C at the same heating rate.

## 3 Results and discussion

### 3.1 Production and purification of bio-based 2,3-BDO

Two batches of 1-L fermentations were carried out according to the procedure described in the experimental section. The production of 2,3-BDO in the batches was 11.96 and 15.12 g L^−1^, respectively. The lower concentration of 2,3-BDO in the first batch resulted from the incomplete utilisation of lactose by the bacterium, with 7.6 g L^−1^ of lactose remaining unused, while in the second batch, lactose was completely used.

There are not many reports regarding the use of lactose or whey to produce 2,3-BDO by fermentation, and most of them involve pathogenic bacteria. In addition, natural 2,3-BDO producers usually exhibit unsatisfactory fermentation performance when lactose or whey is used as a substrate, thus requiring the implementation of extensive metabolic engineering to improve it. Only one relevant study dealing with the production of 2,3-BDO by *L. lactis* from whey is found in the literature, reporting the production of up to 51 g/L 2,3-BDO (Kandasamy et al., 2016). However, they used extensively engineered strains and a lactose concentration that was double the amount used in this work. So, as far as we know, *L. lactis* 43103 is the non-pathogenic and non-engineered bacterial strain, showing the best performance in producing 2,3-BDO from whey (Roncal et al., 2023).

The broths of both fermentation batches were independently subjected to the purification process, resulting in the recovery of 8.55 and 11.72 g of 2,3-BDO, respectively. The recovery yields of 2,3-BDO in both batches were 75.3% and 79.5%, respectively. There were three main steps in which 2,3-BDO losses occurred: i) centrifugation, where a fraction of liquid (containing 2,3-BDO) remains associated with the cell sediment; ii) ultrafiltration, where approximately 10% of liquid volume remains in the retentate fraction; and iii) extraction, where, following the two extractions, approximately 10% of 2,3-BDO remains unextracted in the broth. Considering the points where these losses occur and that they are hardly avoidable, it seems difficult to significantly improve the yield result.

**FIGURE 3:**
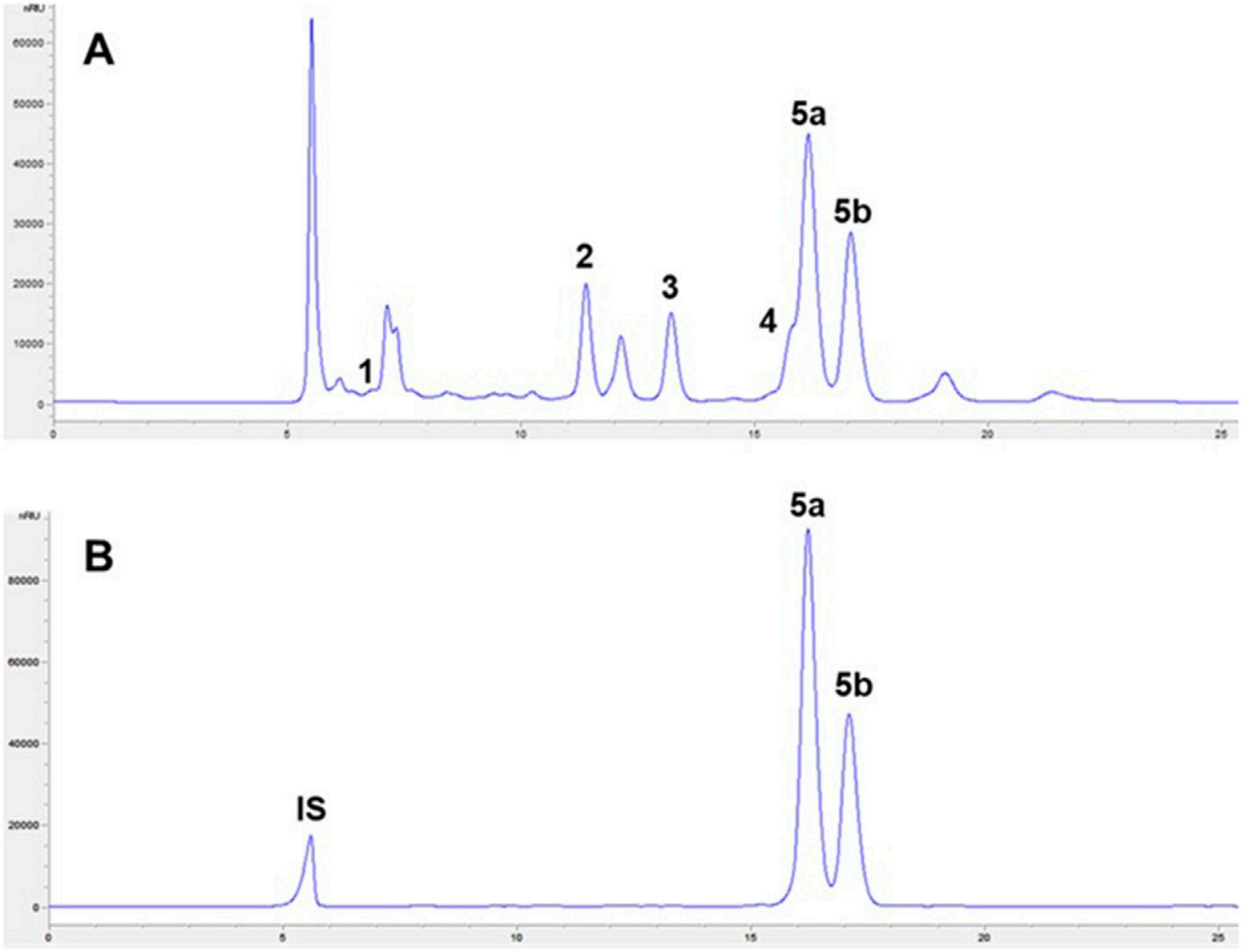
HPLC chromatograms of samples of the original fermentation broth **(A)** and purified 2,3-BDO **(B)** from the second batch. Peaks: 1, lactose; 2, lactic acid; 3, acetic acid; 4, acetoin (shoulder close to peak 5a); 5a, R,R-2,3-BDO; 5b, meso-2,3-BDO; and IS, internal standard.

The purity of purified 2,3-BDO was high, close to 90% in both batches. Purity was calculated as the ratio of 2,3-BDO mass quantified by HPLC analysis to the weighted total mass of the purified product. A comparison of the chromatograms of samples of the original fermentation broth of the second batch and resulting purified 2,3-BDO is shown in Figure 3 (for the first batch, they are identical). In the chromatogram of the purified product, only the peaks of 2,3-BDO were detected (the other peak is the internal standard). 2,3-BDO shows two peaks in chromatograms, corresponding to the R,R- (left) and meso-isomers (right) (Roncal et al., 2023). The petroleum-based 2,3-BDO counterpart was a mixture of R,R-, S,S-, and meso-isomers.

A summary of the whole process of 2,3-BDO production and purification is shown in Table 2.

**TABLE 2:** Summary of the whole process of 2,3-BDO production and purification.

Parameter	Batch 1	Batch 2
Broth volume (mL)	949	975
[2,3-BDO] in broth (g L-1)	11.96	15.12
Total 2,3-BDO (g)	11.35	14.74
Purified 2,3-BDO (g)	8.55	11.72
Recovery yield (%)	75.3	79.5
Purity (%)	89.7	92.7

The produced two batches demonstrated the reproducibility of the process. Batch 2, with a higher yield and purity, was selected for synthesising water-based polyurethanes.

### 3.2 Waterborne PUDS

**FIGURE 4:**
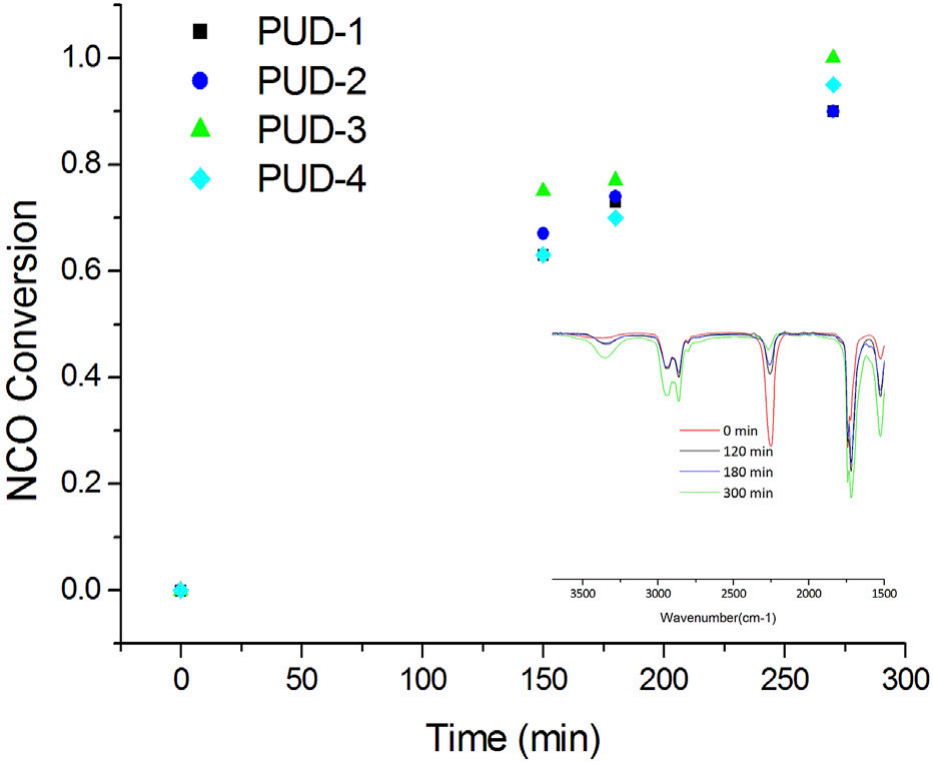
NCO conversions in PUD synthesis; FTIR spectra during PUD-3 synthesis.

The reactivity of the two series of PUDs was analysed by monitoring the conversion of NCO groups by infrared spectroscopy. The presence of 2,3-BDO as a chain extender in the last synthesis stage did not have a significant effect on the NCO conversion rate, and in all petroleum-based and bio-based cases, the conversion of NCO groups was similar for the same tested reaction conditions (regardless of isomerisation). Furthermore, the transition from IPDI and polycarbonate diol to bio-based PUDs with lysine-derived isocyanate, bio-based polyether polyols, and a co-solvent, did not result in any significant variation in the conversion rate throughout the different stages (pre-polymerisation, neutralisation, and extension) of the synthesis process (Figure 4).

**FIGURE 5:**
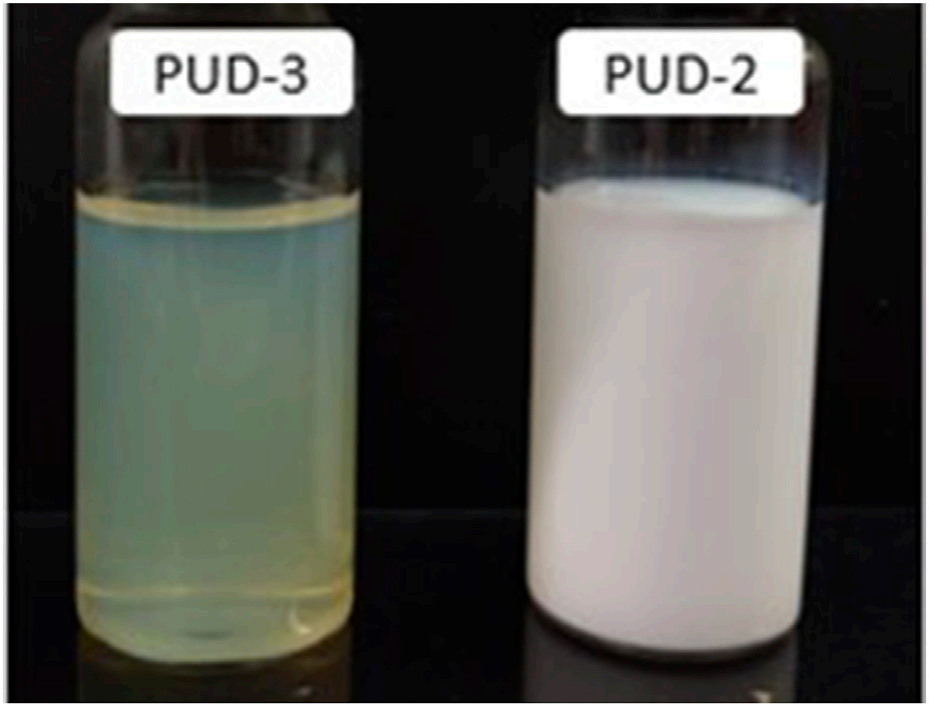
Images of PUDs.

The DLS results revealed that every dispersion had a monomodal size distribution with a very low polydispersity (PDI), regardless of the chain extender and the overall composition of the polyurethane dispersion. In petroleum-based dispersions, the substitution of 1,4-BDO with 2,3-BDO led to an increased particle size. A shorter-chain hydrocarbon between the reactive hydroxyl groups in 2,3-BDO involved stiffening of the hard segment. Furthermore, lateral methyl substituents within the structure, together with the enhanced stiffness within the hard segment, seemed to hinder the packing of the polyurethane chains in nanoparticles for equal concentration of the internal emulsifier and average molecular weight (Yang and Cui, 2022; Garca-Pacios et al., 2010). In lysine-based PUDs, more flexible isocyanate chains led to reduced average particle size in comparison to petroleum references, which could also be favoured by the previously demonstrated reduction in the particle size from higher-viscosity of CY as a co-solvent (Germán et al., 2021) (see Figure 5). In these systems, the lower molecular weight of the polyol in the soft segment, coupled with a higher content of rigid segments and associated stiffness of Velvetol H500, resulted in a larger particle size than that for Velvetol H1000. Nevertheless, the analysis of electrostatic stabilisation, evaluated by measuring Z potential, demonstrated excellent stability from values below −30 mV (Lowry et al., 2016) in all cases, regardless of the dispersion particle size (Table 3).

**TABLE 3:** Particle size and stability of PUDs.

Polyurethane	Particle size distribution DLS				Z potential (mV)
	PDI	d_10_ ^a^ (nm)	d_50_ ^a^ (nm)	d_90_ ^a^ (nm)	
PUD-1	0.10	58.66	86.23	126.82	−36.82
PUD-2	0.08	169.38	235.66	330.28	−42.12
PUD-3	0.12	30.08	46.15	70.82	−55.92
PUD-4	0.12	63.25	97.86	151.61	−51.49

^a^ dn: n% of particles having a diameter below this value.

**FIGURE 6:**
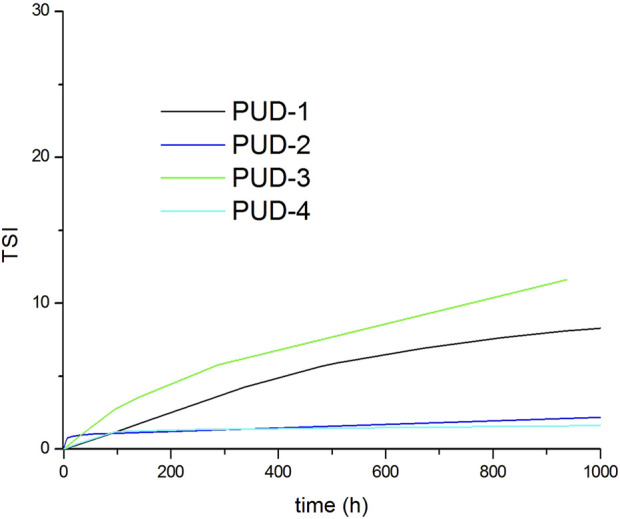
TSI destabilization of synthesised PUDs.

The study of medium-term stability through TSI calculation demonstrated that all PUDs presented certain instability, which evolved asymptotically over time (Figure 6). This destabilisation process was characterised by a low slope of the kinetics curves, indicating a significantly slow destabilisation process, with values of TSI below 4 in the first 6 days. This is very useful for maintaining the properties of the dispersion over long periods, ensuring proper storage and enabling further formulation activities. In all cases, these slight destabilisation processes were not detected by the naked eye, keeping a homogeneous appearance after 40 days despite composition (Table 4).

**TABLE 4:** Long-term stability data (TSI data).

Polyurethane	1 day	6 days
PUD-1	1.3	2.4
PUD-2	0.9	1.1
PUD-3	0.7	3.6
PUD-4	0.3	1.2

Both Z potential and TSI values describe polyurethane dispersions that are stable and homogeneous over the tested time, which is a critical requirement for the formulation of PUD-based coatings and adhesives.

The analysis of the molecular weight confirmed negligible differences in the polymerisation degree from petroleum-based 2,3-BDO as a chain extender, with just a slight increment in polydispersity (Table 5). The results indicated that the chain extenders with different structures affected polydispersity only to a limited extent, due to secondary hydroxyl groups and methyl-related steric hindrance, but within a range that is reasonable for segmented polyurethanes (Ling et al., 2016). Similar molecular weight was achieved for bio-based systems, with just a slight reduction in the dispersion based on a polyether polyol with a shorter-chain length (H500). The slightly remarkable difference in bio-based PUDs was a broader molecular weight distribution from the polyurethane composition, as described in the literature when L-Lysine ethyl ester diisocyanate is used (Marcos-Fernández et al., 2006).

**TABLE 5:** Molecular weight and thermal properties of synthesised PUDs.

Polyurethane	GPC		T_g_ (°C)
	Molecular weight (g mol^-1^)	PDI	
PUD-1	24,382	1.1	−14.2
PUD-2	27,665	1.7	−14.4
PUD-3	29,604	2.4	−42.2
PUD-4	19,904	2.4	−29.0

**FIGURE 7:**
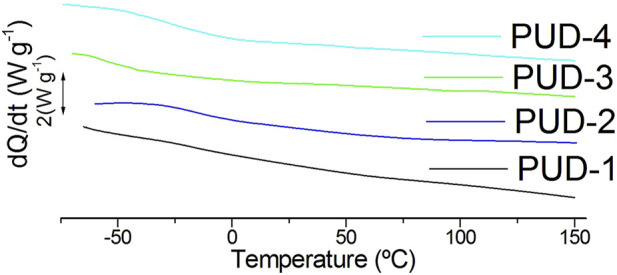
Second heating scan of the DSC analysis for the PUD series.

As observed in Table 5 and Figure 7, the calorimetric analysis demonstrated that the polyurethanes were amorphous polymers with different glass transition temperatures (T_g_) depending on their composition, although the range of obtained T_g_s was in accordance with the standard PUDs. The thermal transition temperature in petroleum-based polymers was not significantly affected by the presence of 2,3-BDO in the hard segment. Furthermore, the use of polycarbonate polyol and the rigid cyclic structure of IPDI in these polyurethanes led to higher T_g_ due to the higher glass transition temperature of components, improved phase miscibility, and greater structural homogeneity than those of bio-based PUDs. In these last systems, where lysine isocyanate is more flexible and polyether polyols are characterised by lower glass transition temperatures and higher flexibility than polycarbonate counterparts, higher free volume and mobility of polymeric segments involved a reduced T_g_ (Asensio et al., 2023). In addition, an observed separation of soft segments from hard segments (see thermogravimetric analysis below) also promoted the reduction of the glass transition temperature in the bio-based solutions (only the glass transitions of the soft segments were visible, and the glass transitions of the hard segments were not visible) (Lee et al., 1994; Puszka et al., 2024). Finally, in bio-based polyurethanes, T_g_ increased from incorporating lower molecular weight 1,3-poly-propanediol in the soft segment for PUD-4.

The TGA analysis of the PUD samples showed that the onset degradation temperature was similar for all the formulations. Then, the variations in components did not influence the thermal stability of the polymers (slightly lower for bio-based formulations with well-defined phases). However, different thermal degradation processes were observed as a function of the composition (Table 6). In IPDI-based polyurethanes, a simultaneous decomposition of hard and soft segments occurred (generally at higher temperatures than for well-separated hard segments) (Ristić et al., 2023). These formulations were characterised by poor phase separation of hard and soft segments, regardless of the chain extender. Therefore, apart from the removal of residual and hydrogen-bonded water (110°C–200°C), similar thermal degradation profiles could be observed, with a main degradation stage at 339°C for PUD-1 and 334°C for PUD-2.

**TABLE 6:** Thermal stability of polyurethane synthesised: T5% is the temperature of 5% mass loss, T max1 (°C) is the first maximal degradation temperature, and T max2 (°C) is the second maximal degradation temperature.

Samples	T 5% (°C)	T max1 (°C)	% weight loss	T max2 (°C)	% weight loss
PUD-1	245	339	43		
PUD-2	264	334	54		
PUD-3	229	278	15	432	73
PUD-4	215	277	17	428	72

**FIGURE 8:**
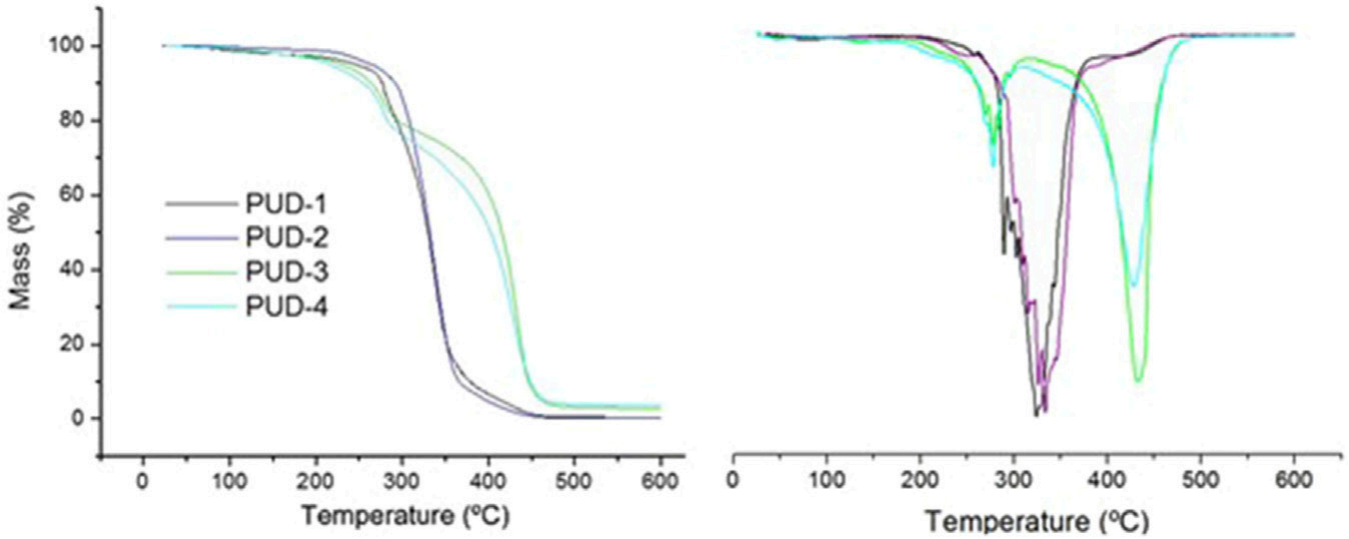
Thermogravimetric analysis of synthesised polyurethane with different compositions.

These derivative curves of thermogravimetric analysis in Figure 8 clearly demonstrated a pronounced separation of hard and soft phases in bio-based polyurethanes from distinct degradation stages (Wang and Hsieh, 1997). In this case, the use of different molecular weight polyether polyols in bio-based polyurethanes did not show significant differences in this degradation profile. Then, an equal degradation process of polyurethane bonds was observed at 278°C and 277°C, and a similar degradation stage of soft segments was observed at 432°C and 428°C for PUD-3 and PUD-4, respectively. These results confirmed the different phase-separation structures from petroleum to bio-based polyurethanes and the main influence of the bio-based isocyanate on the associated thermal degradation profile, which was demonstrated by Brzoska et al. (2024).

The FTIR spectra of the corresponding polyurethanes were characterised by the same relevant bands corresponding to N–H stretching band at 3,325 cm^−1^ and CN–NH stretching band at 1,527 cm^−1^. There were not any significant differences from 2,3-BDO as a chain extender, but just slight differences from the bio-based isocyanate and polyol.

**FIGURE 9:**
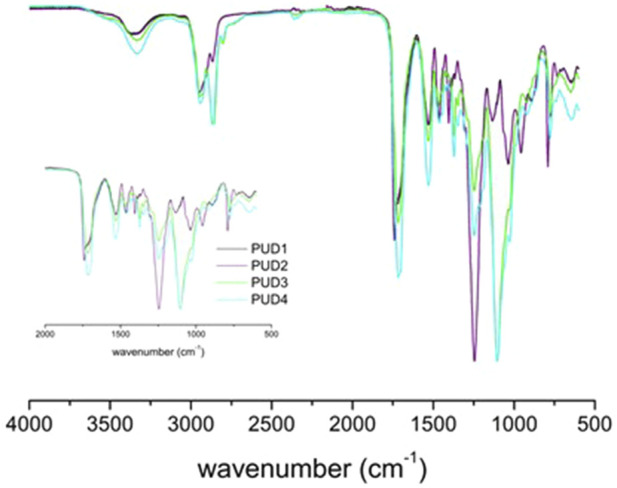
FTIR spectra of synthesised polyurethanes.

The analysis of the spectra in the carbonyl stretching region allowed for identifying a clearer urethane hydrogen bond interaction from the signal at a lower wavenumber than that of free carbonyl in the case of petroleum-based structures (H-bonded urethane at 1,716 cm^−1^). It is worth mentioning that petroleum-based polyurethane spectra showed a urethane peak (1,739 cm^−1^) and hydrogen-bonded urethane peak (1,716 cm^−1^), while in bio-based polyurethane, only H-bonded urethane at 1,716 cm^−1^ is observed. The predominant hydrogen bonds present in bio-based polyurethane, as shown in Figure 9, favoured the formation of microphase separation, as reported by Cheng et al. (2022). This was in accordance with the identified poorer phase separation of hard and soft segments for IPDI-based polyurethanes from thermal analysis related to the degradation profile and lower thermal mobility of polymer chains.

## 4 Conclusion

High-purity (90%) 2,3-BDO was produced through the fermentative valorisation of whey (greater than 75% yield) using *L. lactis* 43103. The feasible use of this renewable diol as a chain extender in high bio-based content PUDs was demonstrated. In addition, no significant effect on the energy expenditure required for the synthesis process, reaction kinetics, and dispersion capacity was identified.

The study revealed that using 2,3-BDO as a chain extender resulted in an increased particle size distribution of the final water dispersion compared to 1,4-BDO. However, both petroleum-based and bio-based dispersions presented a particle size distribution below 400 nm and electrostatic stability (Z potential below −30 mV) for the identical emulsifier system. The long-term stability evaluation did not show any considerable difference in PUDs for 40 days of analysis.

The molecular weight of the polyurethanes was only affected by the reduction in the molecular weight of the used bio-based polyol. Furthermore, the lower T_g_ value in bio-based polymers resulted from the associated different polyurethane structure when incorporated lysine-derived isocyanate and polyether polyols. This glass transition temperature in bio-polyurethanes was also strongly affected by the chain length in the soft segment. In addition, according to TGA and FTIR analysis, this corresponding different polymer structure resulted in a remarkable phase separation in bio-based polyurethanes, not produced for petroleum-based PUDs, just characterised by a single degradation stage in thermal degradation.

In conclusion, a high-quality polyurethane dispersion with a bio-based content greater than 80%, derived from renewable components, was achieved using fermentation-derived 2,3-BDO as a chain extender by the prepolymer missing process. Renewable and biodegradable dihydrolevoglucosenone (CY) was used as the reaction medium (co-solvent) to produce more sustainable waterborne processes and products containing bio-based 2,3-BDO.

## Data Availability

The raw data supporting the conclusions of this article will be made available by the authors, without undue reservation.
